# Brazilian Propolis Intake Decreases Body Fat Mass and Oxidative Stress in Community-Dwelling Elderly Females: A Randomized Placebo-Controlled Trial

**DOI:** 10.3390/nu15020364

**Published:** 2023-01-11

**Authors:** Miho Kanazashi, Tadayuki Iida, Ryosuke Nakanishi, Masayuki Tanaka, Hiromi Ikeda, Naomi Takamiya, Noriaki Maeshige, Hiroyo Kondo, Tomohiko Nishigami, Toshihide Harada, Hidemi Fujino

**Affiliations:** 1 Department of Physical Therapy, Faculty of Health and Welfare, Prefectural University of Hiroshima, Mihara 723-0053, Japan; 2 Department of Physical Therapy, Faculty of Rehabilitation, Kobe International University, Kobe 658-0032, Japan; 3 Department of Physical Therapy, Faculty of Health Sciences, Okayama Healthcare Professional University, Okayama 700-0913, Japan; 4 Department of Rehabilitation Science, Kobe University Graduate School of Health Sciences, Kobe 654-0142, Japan; 5 Department of Food Science and Nutrition, Nagoya Women’s University, Nagoya 467-8611, Japan

**Keywords:** supplementation, body composition, adiponectin, oxidative stress, antioxidant

## Abstract

This study aimed to investigate the effects of Brazilian propolis on body fat mass and levels of adiponectin and reactive oxygen species among community-dwelling elderly females. This was a double-blind randomized placebo-controlled trial. Altogether, 78 females aged 66–84 years were randomly assigned to the propolis (PRO; *n* = 39) or placebo (PLA; *n* = 39) group. For 12 weeks, the PRO group were given three capsules containing 227 mg of propolis twice a day. Meanwhile, the PLA group were given daily placebo capsules. Of 78 participants, 53 (PLA group: *n* = 28, PRO group: *n* = 25) completed the study. Although no changes were observed in absolute or relative fat mass in the PLA group, they showed a significant decline in the PRO group. The level of serum adiponectin in the PLA group did not change, although that of the PRO group significantly increased. The level of d-ROMs in the PLA group significantly increased, whereas that of the PRO group significantly decreased. The serum SOD activity in the PLA group significantly decreased, whereas that of the PRO group tended to increase. These results suggest that propolis supplementation may decrease body fat mass and oxidative stress among community-dwelling elderly females.

## 1. Introduction

Females undergoing the menopause experience a considerable change in metabolic activity [1]. Postmenopausal females present with a remarkably higher incidence of visceral obesity than that presented by premenopausal females [2]. In elderly females, fat accumulation is accelerated by not only age-related declines in muscle mass and physical activity, but also by changes in carbohydrate and lipid metabolism patterns associated with lower levels of sex steroid hormones caused by the menopause [1]. Fat accumulation can result in obesity. This, in turn, causes different metabolic complications, such as type 2 diabetes and cardiovascular diseases [1], consequently resulting in severe limitations to daily activities and higher mortality rates. Therefore, decreasing fat accumulation is important for maintaining health among elderly postmenopausal females.

Body fat mass shows a positive correlation with systemic oxidative stress in clinically healthy middle-aged females [3]. Oxidative stress is enhanced by increased adipose tissues associated with the menopause [1]. Therefore, postmenopausal females are at a high risk of oxidative stress caused by increased adipose tissues. Different cytokines are secreted from adipose tissues [4], which are involved in the increase or decrease in oxidative stress. The level of circulating adiponectin, which is a favorable adipokine, unlike others, decreases with increased adipose tissues [5,6]. Moreover, it enhances the antioxidant capacity via mitochondrial biogenesis and leads to a higher expression of antioxidant enzymes [7,8]. Therefore, the effects of changes in fat mass on oxidative stress can be partially explained by adiponectin levels.

Exercise and dietary restrictions are effective in controlling fat accumulation. However, since these countermeasures can be difficult to continue, alternative methods should be considered. Daily nutritional supplementation has been found to be effective against obesity and metabolic complications [9,10]. Recently, the effects of polyphenols have been comprehensively assessed in clinical trials in humans [11]. Propolis, a substance produced by bees from the resin collected from trees and shrubs, has long been used as a nonpharmacologic agent [12]. In particular, a growing body of evidence has shown that Brazilian green propolis is beneficial to health because it has different bioactive activities [13]. Previous studies have revealed that Brazilian propolis decreases visceral fat accumulation in ob/ob mice with high fat diet-induced obesity [14] and type 2 diabetes [15]. Therefore, further studies must be conducted to verify the effects of Brazilian propolis on fat mass among humans. To date, numerous studies have examined the therapeutic effects of propolis of various origins among patients with diabetes and other diseases [16,17,18,19]. However, the effect of propolis intake on body fat mass among community-dwelling healthy females remains unknown.

Based on previous reports, we hypothesized that Brazilian propolis supplementation would reduce fat mass and promote adiponectin secretion among females, thereby optimizing an integrated balance between reactive oxygen species (ROS) and antioxidants. This randomized, double-blind, placebo-controlled trial aimed to investigate the effects of ethanol extracts of Brazilian green propolis on fat mass, adiponectin secretion, and oxidative stress among community-dwelling elderly females. This study could contribute to the development of novel therapeutic strategies for preventing obesity and its associated metabolic disorders, such as vascular disease and Alzheimer’s disease, among elderly females.

## 2. Materials and Methods

### 2.1. Participants

**Figure 1:**
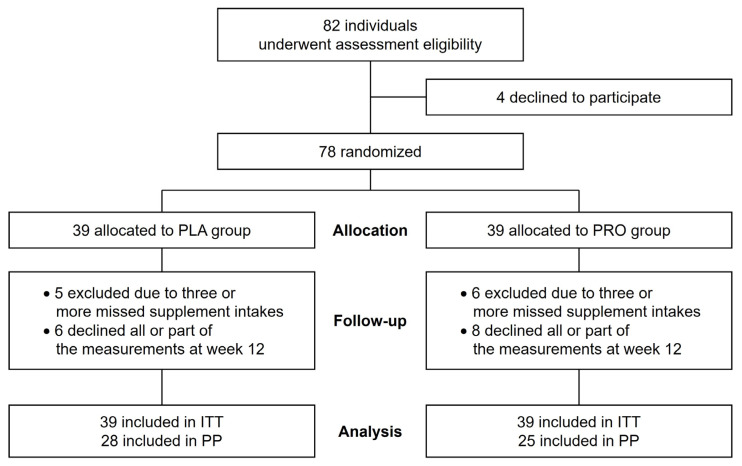
CONSORT flowchart. PLA, placebo; PRO, propolis; ITT, intention-to-treat; PP, per-protocol. Obtained from honeybee hives and extracted using ethanol. Wheat germ oil is extracted by pressing the wheat germ and is used as a solvent for propolis supplements. Therefore, it was selected as a placebo in this study. The supplements used in the present study are commercially available as health foods. As for the dosage of propolis, an animal toxicity study has shown that the non-observed effect level is 1400 mg/kg/day [20]. In reports on human subjects, 400–900 mg/day of propolis has been applied to patients with rheumatoid arthritis [21], type 2 diabetes mellitus [22], or COVID-19 [23], and elderly people (average 72.8 years) [24]. In the present study, 454 mg/day of propolis was administered to the subjects and none of them complained of side effects. This suggests that propolis and its dosage used in the present study are likely to be safe.

Healthy postmenopausal elderly females were recruited as trial volunteers for this study via the use of a public relations magazine in Mihara City, Japan. The inclusion criteria were healthy, postmenopausal females aged 65 years or older who were independent in their daily lives. Meanwhile, the exclusion criteria were females with orthopedic diseases, psychiatric disorders, and dementia; those on medication; those taking other supplements; and those who missed at least 3 days of supplementation during the 12-week intervention period. The participants received a detailed explanation regarding the contents and methods of this study before the trial began. Written informed consent was obtained from 82 volunteers. Among them, 78 attended the baseline examination session and were randomly assigned to either the propolis or the placebo group. The participants were provided with individual identification numbers to ensure anonymity. After matching according to age (±3 years) and body mass index (BMI; ± 3 kg/m2), the participants were divided into the group receiving propolis capsules (PRO; *n* = 39) and the group receiving placebo capsules (PLA; *n* = 39). The authors were blinded to the information regarding the groups to which the participants were assigned. Eleven participants (PRO group, *n* = 6; PLA group, *n* = 5) dropped out of the study because they missed taking the supplementation more than three times during the 12-week intervention period. In addition, 14 participants (PRO group, *n* = 8; PLA group, *n* = 6) who did not participate in the measurement after the 12-week intervention or declined some measurements were excluded from the analysis. Finally, we analyzed 25 and 28 participants in the PRO and PLA groups, respectively. Figure 1 shows the sampling scheme of this study.

### 2.2. Study Design

This was a randomized, double-blind, and placebo-controlled trial. The PRO group were given three soft capsules containing 227 mg/3 capsules of propolis twice per day, i.e., after breakfast and dinner (total intake: 454 mg/6 capsules/day) for 12 weeks. Meanwhile, the PLA group were given placebo capsules at the same dosage and frequency for 12 weeks. The participants were instructed to take the capsules with water or tea. Furthermore, all participants were required to not alter their lifestyle habits, such as diet and exercise, during the study period. To assess compliance, the participants were instructed to record changes in their lifestyle and health status and daily capsule intake.

The study was conducted in accordance with the Declaration of Helsinki and approved by the Institutional Review Board (or Ethics Committee) of the Prefectural University of Hiroshima (15MH064) and Kobe University Graduate School of Health Sciences (277-2). Furthermore, the trial was registered at the University Hospital Medical Information Network (UMIN) Center (ID: UMIN000020459). Informed consent was obtained from all subjects involved in the study.

### 2.3. Brazilian Propolis Supplementation

We prepared 227 mg/3 capsules of propolis supplement using Propolis 300^®^ (Yamada Bee Farm, Okayama, Japan), which is a commercial product, and the placebo supplement by replacing propolis with wheat germ oil. The raw materials for propolis supplementation include gelatin, propolis extract, glycerin fatty acid ester, glycerin, arginine, pectin, and cocoa color. The propolis extract was mainly composed of flavonoids

### 2.4. Assessment Procedures

All assessments were performed by a physician, radiologist, nurse, dietitian, and ten physical therapists. The assessments before and after the intervention lasted for approximately 60 min. They were conducted at the Mihara Campus of Prefectural University of Hiroshima using a standardized protocol by the same researcher.

All assessments were conducted between 09:00 and 15:00 in the same laboratories. The participants were instructed to fast for 2 h before the assessments. The consumption of Japanese tea was allowed before and during the assessments to prevent dehydration. Upon arrival, the participants were instructed to sit. Next, their blood pressure and heart rate were evaluated and their health was examined by the physician. Their body mass was measured at increments of 0.1 kg using a calibrated digital scale and their height was evaluated at increments of 0.1 cm using a wall-mounted height meter. Body mass index (BMI) was calculated as weight (kg) divided by squared height (m^2^). With reference to the participant’s lifestyle and other records, dietitians and physical therapists interviewed the participants about their lifestyle habits, including diet and exercise, during the intervention period.

While in the sitting position, the nurse collected blood samples from a superficial vein in the forearm of all patients via venipuncture. Approximately 4 mL blood was collected and placed into prechilled silicone-coated and lithium heparin-coated tubes. A portion of the blood sample was used to measure the level of diacron-reactive oxygen metabolites (d-ROMs), which is an indicator of blood oxidative stress, using the FREE Carrio Duo (WISMERLL Co., Tokyo, Japan) as per the manufacturer’s instructions. The level of d-ROMs was expressed in Carratelli units (U.CARR) [25] (normal range: 250–300 U.CARR), where 1 U.CARR corresponds to 0.08 mg/dL of H_2_O_2_. To collect serum, the remaining blood sample was centrifuged at 3000× *g* for 15 min at 4 °C. The collected serum was stored in a deep freezer at −80 °C until subsequent biochemical analysis. Antioxidative superoxide dismutase (SOD) activity and adiponectin concentration were evaluated using the serum sample. SOD activity was assessed using the SOD Assay Kit-WST (S311, Dojindo, Kumamoto, Japan) by employing the colorimetric quantification method as per the manufacturer’s instructions. The adiponectin concentration was evaluated using the commercial ELISA kit (Human Total Adiponectin/Acrp30 Quantikine ELISA Kit/514-96041, DRP300, R&D Systems, Minneapolis, MN, the USA) as per the manufacturer’s instructions.

Body composition and bone mineral density (BMD) were assessed by a radiologist using Discovery (Hologic, Inc., Bedford, MA, USA) with dual-energy X-ray absorptiometry (DXA), which is a highly accurate method [26,27]. Absolute and relative fat mass and absolute lean body mass, except that of the head, were evaluated. The BMD of the lumbar spine (L2–L4) and the left femur was evaluated.

To evaluate hand grip and knee extension strength, which are significantly correlated with the total body skeletal muscle mass, skeletal muscle function was assessed by physical therapists. The hand grip strength of the dominant hand was analyzed to the nearest 0.5 kg using a hydraulic hand dynamometer (T.K.K. 5401; Takei Scientific Instruments Co., Ltd., Niigata, Japan) while the participants remained in a standing position and the upper limbs remained in a drooping position. Evaluations were conducted twice at intervals of 1 min, and the best score was recorded as the hand grip strength. The knee extension strength of the dominant leg was evaluated using a seated measuring device (Isoforce GT-330, OG Giken, Okayama, Japan). The measurements were conducted while the participants were sitting in a chair with the backrest tilted back at 12° from upright position, with two belts on the thighs and one on the waist, and with the knee joint flexed at 90°. Measurements were conducted at intervals of 2 min, and the best score was recorded as the knee extension strength. During the measurements of hand grip and knee extension, the participants were instructed not to stop breathing to prevent the occurrence of the Valsalva effect.

### 2.5. Statistical Analysis

All continuous data were presented as mean standard error of the mean or median (interquartile range) for parametric or nonparametric continuous variables, respectively. The baseline characteristics between the two groups were compared using a Student’s unpaired *t*-test or a Mann–Whitney *U*-test for parametric or nonparametric continuous variables, respectively. All 78 subjects were analyzed using mixed-effects models for repeated measures with intervention, study time point, and their interaction according to intention-to-treat (ITT). As per-protocol (PP) analysis, 53 subjects were using a two-factor, repeated-measures analysis of variance with time as the within-participant factor and group as the between-participant factor. If there was a significant time × group interaction, post hoc analyses with Bonferroni correction were performed to identify significant within-group effects. Intragroup changes in the levels of blood markers before and after the intervention were compared using the Wilcoxon rank-sum test. For all statistical analyses, a *p* value of <0.05 was considered statistically significant. All statistical analyses and graphical data representations were performed using IBM SPSS Statistics 27 (IBM Japan, Ltd., Tokyo, Japan) and Prism version 7 (GraphPad Software Inc, San Diego, CA, USA).

The sample size calculation and power analysis were performed as follows: First, we estimated a treatment effect of 1 kg and standard deviation of 5 kg on fat mass and calculated effect size before study. Next, we calculated the sample size using the G*Power 3.1 software (Heinrich-Heine-Universität Düsseldorf) for MAC. A two-way repeated measures ANOVA indicated that a total sample size of 52 was needed to reach 80% power, in order to detect the interaction effect size of 0.20 at a significance level of 0.05.

## 3. Results

Table 1 shows the baseline characteristics of elderly females in each group. No significant differences were noted in any measurement between the two groups. Based on lifestyle and health records during the experimental period, no change was observed in the participant’s lifestyle habits, such as exercise and diet.

**Figure 2:**
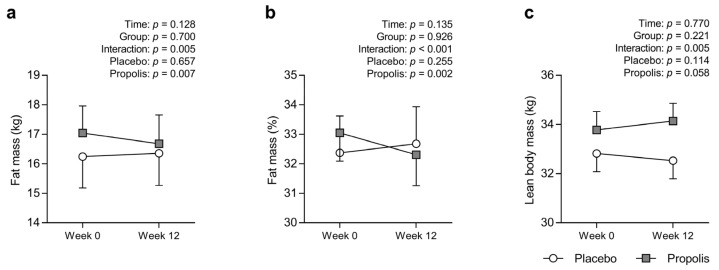
Absolute fat mass (**a**), relative fat mass (**b**), and absolute lean body mass (**c**), with the exception of the head in the placebo (white) and propolis (gray) groups. Values were presented as mean ± standard error of the mean. Variables were analyzed via two-factor repeated-measures analysis of variance, with time (week 0 vs. 12) and group (placebo vs. propolis) as factors. A *p* value of <0.05 was considered significant. If there was a significant time × group interaction, post hoc analyses with Bonferroni correction were performed to identify significant within-group effects.

An interaction effect was observed for absolute and relative fat mass (*p* = 0.005 and *p* < 0.001, respectively; Figure 2a,b), i.e., the absolute and relative fat mass in the PLA group did not change (pre: 16.2 ± 1.1 kg, post: 16.4 ± 1.1 kg, *p* = 0.657; pre: 32.4% ± 1.2%, post: 32.7% ± 1.2%; *p* = 0.255, respectively). Meanwhile, those of the PRO group significantly decreased (pre: 17.0 ± 0.9 kg, post: 16.7 ± 1.0 kg, *p* = 0.007; pre: 33.1% ± 1.0%, post: 32.3% ± 1.1%, *p* = 0.002, respectively). An interactive effect was observed for absolute lean body mass (*p* = 0.005; Figure 2c), i.e., the absolute lean body mass in the PLA group did not change (pre: 32.8 ± 0.7 kg, post: 32.5 ± 0.7 kg, *p* = 0.114). However, that of the PRO group tended to increase (pre: 33.8 ± 0.7 kg, post: 34.1 ± 0.7 kg, *p* = 0.058).

There was no significant change between body mass, BMI, lumbar spine, proximal femur BMD, hand grip strength, or knee extension strength before and after the intervention (Table 2).

**Figure 3:**
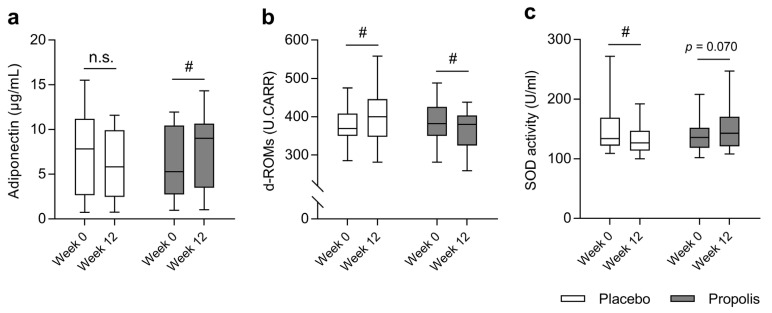
Boxplots showing values of serum levels of adiponectin (**a**), blood levels of d-ROMs (**b**), and serum SOD activity (**c**) before and after the intervention in the placebo (white) and propolis (gray) groups. Boxplots show the median, upper and lower quartiles, and maximum and minimum values. The differences between before and after the intervention in each group were compared using a Mann–Whitney *U*-test. A *p* value of <0.05 was considered significant. # Significantly different between before and after the intervention. d-ROMs, diacron-reactive oxygen metabolites; SOD, superoxide dismutase.

The level of serum adiponectin in the PLA group did not change after the intervention (pre: 7.9 (3.3–11.1) μg/mL, post: 5.8 (2.5–9.9) μg/mL; *p* = 0.145), but significantly increased in the PRO group (pre: 5.3 (2.8–10.0) μg/mL, post: 9.0 (3.8–10.6) μg/mL; *p* = 0.007; Figure 3a). The blood level of d-ROMs in the PLA group significantly increased (pre: 374.5 ± 8.9 U.CARR, post: 402.4 ± 12.5 U.CARR; *p* < 0.001), but significantly decreased in the PRO group (pre: 385.6 ± 111.8 U.CARR, post: 365.7 ± 10.5 U.CARR; *p* = 0.013; Figure 3b). The serum SOD activity in the PLA group significantly decreased (pre: 132 (122–162) U/mL, post: 127 (115–143) U/mL; *p* = 0.015), but tended to increase in the PRO group (pre: 136 (119–152) U/mL, post: 143 and (124–168) U/mL; *p* = 0.070; Figure 3c). None of the participants presented with life-threatening side effects following supplementation with propolis and placebo during the experimental period.

## 4. Discussion

Our prospective interventional study showed that propolis had a positive effect on absolute and relative fat mass, relative lean body mass, and levels of adiponectin and ROS among community-dwelling elderly females. Therefore, propolis supplementation may be an effective strategy for preventing disorders caused by body fat accumulation among elderly females. In previous studies, the anti-obesity and antioxidant properties of propolis have been found to be effective against diseases such as type 2 diabetes [13,14,16,17,18,19,22]. To the best of our knowledge, this is the first study that has shown the efficacy of propolis supplementation among community-dwelling healthy elderly females.

Propolis supplementation caused the absolute and relative fat mass to decrease among elderly females. The DXA method, which is the gold standard for body composition measurements, was used to assess fat mass, and the results of this study were highly reliable. Propolis has been shown to reduce fat mass in obese animal models [14]. The mechanism was correlated with the fact that propolis induces the activation of peroxisome proliferator-activated receptor γ (PPARγ) [13], which causes browning of white adipocytes. Artepillin C, an ingredient derived from propolis, can induce brown-like adipocytes’ formation [28], which increase energy expenditure and promote fat loss. Moreover, decreased fat mass may be associated with the positive effects of propolis on AMP-activated protein kinase (AMPK) in the skeletal muscle [29]. AMPK is a key factor for metabolic activity, including lipid metabolism [30]. Therefore, propolis supplementation may increase skeletal muscle lipid metabolism via AMPK activation, which may also promote fat loss. Taken together, a lower fat mass, as observed in this study, may be accounted for by a higher lipolytic capacity in adipocytes and skeletal muscles. In addition, another possible explanation for reduced fat mass may be attributed to the positive effect of propolis on skeletal muscle mass, resulting in increased energy expenditure through the basal metabolic rate. A previous study that has reported on propolis indicated that propolis has a protective effect on aged skeletal muscle via various biological activities such as anti-inflammatory and antioxidative actions [31]. Indeed, among the elderly females in this study, lean body mass tended to increase following supplementation with propolis. In contrast, hand grip and knee extension strength did not change with or without propolis supplementation. Increased lean body mass is not always accompanied by changes in limb muscle strength [32,33]. In addition, licorice flavonoid oil supplementation consisting of another type of polyphenol increased the trunk muscle mass but not limb muscle mass, with no improvement observed in limb muscle strength in older adults [34]. Therefore, propolis supplementation may have increased lean body mass mainly in the trunk region in this study as well, resulting in higher energy expenditure. Taken together, these results indicate that propolis supplementation is an effective strategy for reducing fat mass, which may prevent obesity-related metabolic complications among elderly females.

Propolis supplementation increased the levels of adiponectin among elderly females. To the best of our knowledge, this is the first study to show that propolis increases the level of adiponectin in humans. Brazilian propolis-derived components have molecular mechanisms that act on adiponectin expression. Previous research has shown that Brazilian propolis is associated with adiponectin expression via the activation of PPARγ in adipocytes [35]. This report supports the results of the present study, i.e., the circulating level of adipokines, including adiponectin, is a good indicator of fat mass and other body properties. Age-related changes in body fat distribution include loss of subcutaneous fat, accumulation of visceral fat, and deposition of ectopic fat [36]. Furthermore, the changes in sex hormones associated with the menopause cause visceral adiposity among elderly females [1]. Visceral fat accumulation shows a negative correlation with the circulating levels of adiponectin [37,38,39]. From these reports, the increased adiponectin level with propolis intake could be also attributed to a possible decrease in visceral fat mass. Therefore, propolis supplementation may have a positive effect on visceral fat accumulation among elderly postmenopausal females.

Oxidative stress, as assessed using blood levels of d-ROMs, decreased with propolis supplementation among elderly females. Brazilian propolis contains different bioactive components, such as artepillin C and baccharin, and has antioxidant effects [40,41,42,43]. Therefore, decreased ROS accumulation in the blood samples of propolis-treated elderly females may be partially attributed to the antioxidant activity of Brazilian propolis per se. However, the activation of PPARγ enhances antioxidative capacity by increasing antioxidant expression, including that of SOD [44,45]. In addition, adiponectin stimulates antioxidant enzyme expression [7]. Therefore, propolis supplementation may enhance antioxidant expression, including that of SOD, as evidenced in the PRO group that showed a tendency of increase in SOD activity. This might have reduced the level of ROS in the blood of the propolis-treated group. Oxidative stress is associated with the pathology of various age-related conditions [46]. Furthermore, postmenopausal females are highly exposed to the effects of ROS owing to a decreased antioxidant capacity and low levels of estrogen [47]. Therefore, the effect of propolis on reducing the risk of oxidative stress may partially prevent oxidative stress-related diseases in postmenopausal aging females.

However, propolis did not affect BMD. Nevertheless, it is premature to conclude that it did not have a protective effect on human bones based on this 12-week intervention study. The estrogenic effects of propolis, which can reduce the development of postmenopausal symptoms, were observed in an ovariectomized animal model [48]. Changes in the levels of estrogen and other sex hormones associated with the menopause can contribute to osteoporosis [49] among elderly females. Therefore, propolis may be effective against osteoporosis among elderly females who have gone through the menopause. However, further research must be conducted to evaluate the appropriate dosage and duration of propolis treatment.

As medical care costs continually rise with the increase in the elderly population, elderly individuals must manage their own health. Fat accumulation can be prevented or decreased to a certain degree via self-management. To reduce body fat mass, regular exercise and dietary restriction are required. However, the lifestyle habits of elderly individuals are difficult to modify. In association with this, the intake of functional foods, including propolis, can be considered without making any major alterations to lifestyle habits and can be a practical and sustainable method.

This study had several limitations. First, we failed to report the food consumption and the levels of physical activity of participants during the study period. We confirmed through interviews that there were no changes in the eating or exercise habits of the participants. However, because the specific quantification of these factors was not performed, their effects cannot be taken into account in this study. Second, we only included elderly females. Therefore, the effects of propolis supplementation on reducing body fat and blood levels of ROS cannot be generalized to males and younger individuals. Third, although body fat was evaluated accurately using the DXA method, the distribution of adipose tissues was not assessed. In particular, visceral and ectopic fats are involved in the development of insulin resistance and vascular disorders [50,51]. Therefore, future studies must be conducted to evaluate the distribution of body fat. Fourth, general blood properties, such as the levels of triglyceride and low-density lipoprotein, were not investigated. These indices are commonly used as indicators of metabolic function, including lipid metabolism. Therefore, they should be evaluated in future studies. Fifth, the data at week 12 should have been obtained for subjects (placebo, *n* = 5; propolis, *n* = 6) who were excluded from the experiment due to missed supplement intakes during the experiment. Since there was no discrepancy between the ITT and PP analyses in fat mass for the primary outcome, the results of the PP analysis were used in this study.

## 5. Conclusions

Propolis supplementation reduced body fat accumulation, increased blood levels of adipokine, and decreased blood levels of ROS among elderly females, which are all beneficial to health. Therefore, propolis may be effective in preventing various disorders, especially those associated with fat accumulation among elderly females.

## Figures and Tables

**Table 1:** Baseline characteristics of the participants in each group.

	PLA (*n* = 28)	Propolis (*n* = 25)	*p* Value
Physical characteristics			
Age (years)	75 (69–78)	75 (72–78)	0.447 *^a^*
Height (cm)	149.7 ± 1.0	151.2 ± 1.1	0.287 *^b^*
Body mass (kg)	53.0 ± 1.6	55.1 ± 1.6	0.368 *^b^*
BMI (kg/m^2^)	23.7 ± 0.7	24.0 ± 0.6	0.716 *^b^*
Body composition			
Fat mass (kg)	16.2 ± 1.1	17.0 ± 0.9	0.576 *^b^*
Fat mass (%)	32.4 ± 1.2	33.0 ± 1.0	0.675 *^b^*
Lean body mass (kg)	32.8 ± 0.7	33.8 ± 0.7	0.368 *^b^*
Bone mineral density			
Lumbar spine (g/cm^2^)	0.80 ± 0.02	0.80 ± 0.03	0.955 *^b^*
Proximal femur (g/cm^2^)	0.70 ± 0.02	0.69 ± 0.02	0.556 *^b^*
Muscle function			
Hand grip strength (kg)	25.1 ± 0.6	25.0 ± 0.6	0.841 *^b^*
Knee extension strength (Nm)	94.0 ± 3.5	92.7 ± 4.1	0.809 *^b^*
Blood markers			
Adiponectin (μg/mL)	7.8 (3.1–11.0)	5.3 (2.8–10.0)	0.675 *^a^*
d-ROMs (U. CARR)	374.5 ± 9.1	385.6 ± 11.8	0.451 *^b^*
SOD activity (U/mL)	134 (122–162)	136 (119–152)	0.539 *^a^*

Data are presented as mean ± standard error of the mean or median (interquartile range). Significant differences were analyzed using the Student’s unpaired *t*-test. A *p* value of <0.05 was considered statistically significant. There was no significant difference between the two groups in any measurements at baseline. BMI, body mass index; d-ROMs, diacron-reactive oxygen metabolites; SOD, superoxide dismutase. *^a^* Derived by Mann–Whitney *U*-test. *^b^* Derived by independent samples *t*-test.

**Table 2:** Measurements before and after 12 weeks of supplementation with propolis and placebo Data are presented as mean ± standard error of the mean. Results from both ITT and PP analysis are shown. Variables were analyzed via two-factor repeated-measures analysis of variance, with time (week 0 vs. 12) and group (placebo vs. propolis) as factors. A *p* value of <0.05 was considered statistically significant. For variables with a significant time × group interaction, post hoc analyses with Bonferroni correction were conducted to identify significant within-group effects. * Significantly different from before the intervention. PP, per-protocol; ITT, intention-to-treat; BMI, body mass index; BMD, bone mineral density.

	Placebo (*n* = 28)		Propolis (*n* = 25)		*p* Value (PP Analysis)			*p* Value (ITT Analysis)		
	Week 0	Week 12	Week 0	Week 12	Time	Group	Interaction	Time	Group	Interaction
Body mass (kg)	53.0 ± 1.6	52.8 ± 1.7	55.1 ± 1.6	54.9 ± 1.5	0.170	0.361	0.830	0.039	0.497	0.368
BMI (kg/m^2^)	23.7 ± 0.7	23.6 ± 0.7	24.0 ± 0.6	24.0 ± 0.6	0.157	0.700	0.755	0.037	0.701	0.328
Fat mass (kg)	16.2 ± 1.1	16.4 ± 1.1	17.0 ± 0.9	16.7 ± 1.0 *	0.128	0.700	0.005	0.384	0.479	0.077
Fat mass (%)	32.4 ± 1.2	32.7 ± 1.3	33.0 ± 1.0	32.3 ± 1.0 *	0.135	0.926	<0.001	0.297	0.440	0.005
Lean body mass (kg)	32.8 ± 0.7	32.5 ± 0.7	33.8 ± 0.7	34.1 ± 0.7	0.770	0.221	0.005	0.941	0.509	0.003
Lumbar spine BMD (g/cm^2^)	0.80 ± 0.02	0.79 ± 0.02	0.80 ± 0.03	0.80 ± 0.04	0.855	0.920	0.569	0.663	0.563	0.496
Proximal femur BMD (g/cm^2^)	0.70 ± 0.02	0.70 ± 0.02	0.69 ± 0.02	0.68 ± 0.02	0.522	0.556	0.989	0.267	0.284	0.664
Hand grip strength (kg)	25.1 ± 0.6	24.6 ± 0.6	25.0 ± 0.6	25.1 ± 0.7	0.491	0.860	0.265	0.203	0.550	0.294
Knee extension strength (Nm)	94.0 ± 3.5	97.1 ± 4.4	92.7 ± 4.1	97.2 ± 4.9	0.043	0.921	0.684	<0.001	0.813	0.498

## Data Availability

The data that support the findings of this study are available from the corresponding author upon reasonable request.
